# Soil carbon storage is related to tree functional composition in naturally regenerating tropical forests

**DOI:** 10.1111/1365-2435.14221

**Published:** 2022-11-10

**Authors:** Abby Wallwork, Lindsay F. Banin, Daisy H. Dent, Ute Skiba, Emma Sayer

**Affiliations:** ^1^ Lancaster Environment Centre Lancaster University Lancaster UK; ^2^ UK Centre for Ecology & Hydrology Penicuik UK; ^3^ Max Planck Institute for Animal Behavior Konstanz Germany; ^4^ Department of Environmental Systems Science Institute of Integrative Biology, ETH Zurich Zurich Switzerland; ^5^ Smithsonian Tropical Research Institute Panama City Republic of Panama

**Keywords:** light‐demanding tree species, litter quality, organic matter, secondary succession, shade tolerance, soil carbon storage, tree functional groups, tropical forest regrowth

## Abstract

1. Regenerating tropical forests are increasingly important for their role in the global carbon cycle. Carbon stocks in above‐ground biomass can recover to old‐growth forest levels within 60–100 years. However, more than half of all carbon in tropical forests is stored below‐ground, and our understanding of carbon storage in soils during tropical forest recovery is limited.
2. Importantly, soil carbon accumulation does not necessarily reflect patterns in above‐ground biomass carbon accrual during secondary forest succession, and factors related to past land use, species composition and soil characteristics may influence soil carbon storage during forest regrowth.
3. Using tree census data and a measure of tree community shade tolerance (species‐specific light response values), we assessed the relationship between soil organic carbon stocks and tree functional groups during secondary succession along a chronosequence of 40‐ to 120‐year‐old naturally regenerating secondary forest and old‐growth tropical forest stands in Panama.
4. While previous studies found no evidence for increasing soil C storage with secondary forest age, we found a strong relationship between tree functional composition and soil carbon stocks at 0–10 cm depth, whereby carbon stocks increased with the relative influence of light‐demanding tree species. Light demanding trees had higher leaf nitrogen but lower leaf density than shade‐tolerant trees, suggesting that rapid decomposition of nutrient‐rich plant material in forests with a higher proportion of light‐demanding species results in greater accumulation of carbon in the surface layer of soils.
5. *Synthesis*. We propose that soil carbon storage in secondary tropical forests is more strongly linked to tree functional composition than forest age, and that the persistence of long‐lived pioneer trees could enhance soil carbon storage as forests age. Considering shifts in tree functional groups could improve estimates of carbon sequestration potential for climate change mitigation by tropical forest regrowth.

Read the free Plain Language Summary for this article on the Journal blog.

## Graphical abstract

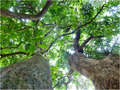

## Resumen

1. Los bosques tropicales en regeneración son cada vez más importantes por su papel en el ciclo global del carbono. Las reservas de carbono en la biomasa aérea pueden recuperarse hasta los niveles de los bosques maduros en un plazo de 60 a 100 años. Sin embargo, más de la mitad de todo el carbono en los bosques tropicales se almacena bajo tierra, y nuestra comprensión del almacenamiento de carbono en los suelos durante la recuperación de los bosques tropicales es limitada.
2. Es importante señalar que la acumulación de carbono en el suelo no refleja necesariamente los patrones de acumulación de carbono en la biomasa aérea durante la sucesión de bosques secundarios y los factores relacionados con el uso pasado del terreno, la composición de especies y las características del suelo pueden influir en el almacenamiento de carbono en el suelo durante la regeneración del bosque.
3. Usando datos del censo de árboles y una medida de la tolerancia a la sombra de la comunidad de árboles (valores de respuesta a la luz específicos de la especie), evaluamos la relación entre las reservas de carbono orgánico del suelo y los grupos funcionales de los árboles durante la sucesión secundaria a lo largo de una cronosecuencia de 40 a 120 años bosques secundarios de regeneración natural y rodales de bosques tropicales primarios en Panamá.
4. Mientras que estudios previos no encontraron evidencia de un aumento del almacenamiento de C en el suelo con la edad del bosque secundario, encontramos una fuerte relación entre la composición funcional de los árboles y las reservas de carbono del suelo a 0–10 cm de profundidad, por lo que las reservas de carbono aumentaron con la influencia relativa de especies de árboles que demanda de luz. Los árboles que requieren luz tenían más nitrógeno en las hojas pero menor densidad de hojas que los árboles tolerantes a la sombra, lo que sugiere que la descomposición rápida del material vegetal rico en nutrientes en los bosques con una mayor proporción de especies que requieren luz da como resultado una mayor acumulación de carbono en la capa superficial de los suelos.
5. *Síntesis.* Proponemos que el almacenamiento de carbono en el suelo en los bosques tropicales secundarios está más fuertemente relacionado con la composición funcional de los árboles que con la edad del bosque, y que la persistencia de árboles pioneros de larga vida podría mejorar el almacenamiento de carbono en el suelo a medida que los bosques envejecen. La consideración de los cambios en los grupos funcionales de los árboles podría mejorar las estimaciones del potencial de secuestro de carbono para la mitigación del cambio climático mediante la regeneración de los bosques tropicales.

## 1 INTRODUCTION

‘Secondary’ or ‘regrowth’ forests make up over half of all remaining tropical forest (Chazdon, 2014) and are therefore increasingly important as key providers of tropical forest ecosystem services, particularly for their crucial contribution to the terrestrial global carbon (C) cycle (Chazdon et al., 2009; Poorter et al., 2016). The C sequestration potential of regenerating tropical forests is substantial, because they rapidly accumulate C in above‐ground biomass during regrowth, for example in a multi‐site chronosequence study in the Neotropics, naturally regenerating tropical forests recovered 90% of old‐growth biomass values after *c*. 66 years (Poorter et al., 2016). However, as much as 60% of the total tropical forest C stock is stored below‐ground in soils (Don et al., 2011) and, in contrast to above‐ground biomass, soil C stocks do not necessarily follow a predictable pattern of accumulation over time during secondary succession (Li et al., 2012; Marín‐Spiotta & Sharma, 2013; Martin et al., 2013; Powers & Marín‐Spiotta, 2017). Important knowledge gaps in our understanding of soil C cycling and storage during secondary forest regrowth limit our ability to accurately predict and manage soil C sequestration in secondary tropical forests, such as the influence of past disturbance versus current land cover (Marín‐Spiotta & Sharma, 2013; Martin et al., 2013). This is further complicated as multiple interacting factors can influence soil C dynamics during forest recovery, including climate (Marín‐Spiotta & Sharma, 2013), soil properties such as pH and nitrogen (N) concentration (Jones et al., 2019), vegetation characteristics such as growth rates, litter production and biomass allocation strategy (Laganière et al., 2010; Lai, 2004) and previous land use (Laganière et al., 2010). However, the relative influence of many of these factors have yet to be quantified (Laganière et al., 2010; Li et al., 2012) particularly in tropical forests, where the high diversity of plant species and differences in tree community functional composition among forests may mask underlying mechanisms.

The extent of soil disturbance during former land use plays a key role in determining initial soil C stocks, but there is nonetheless no clear trajectory of soil C accrual during secondary succession among forests growing on the same former land‐use type (Martin et al., 2013). Importantly, while above‐ground biomass C accumulation over time is a direct result of rapid tree growth during forest recovery (Poorter et al., 2016, 2021), soil C storage depends largely on the interplay between soil physicochemical properties and microbial turnover of organic matter (Cotrufo et al., 2013; Lavallee et al., 2020), which in turn is driven by the quantity and quality of plant inputs (Castellano et al., 2015; Metcalfe et al., 2011).

There is increasing evidence that labile, nitrogen‐rich plant C inputs are particularly important for soil organic matter (SOM) formation, and therefore play a key role in governing rates of soil C accumulation and long‐term C storage (Cotrufo et al., 2019; Hatton et al., 2015; Lavallee et al., 2020). Labile compounds such as sugars and amino acids are more efficiently used by soil microbes than compounds such as lignin, and the resulting microbial products of decomposition become increasingly stabilised by soil minerals (Cotrufo et al., 2013; Liang et al., 2019). As the quality (leaf chemical composition and root exudates) and quantity of plant inputs are determined by tree community structure and composition, changes in forest vegetation during secondary succession should therefore strongly influence soil C dynamics and storage in regenerating tropical forests (Laird‐Hopkins et al., 2017). However, the majority of studies linking litter quality to soil C storage have been conducted in temperate systems (e.g. Chen et al., 2020; Freschet et al., 2013), in part, because it is hard to account for the high diversity of tropical forests in experiments (Clarke et al., 2017). Consequently, we are only just beginning to understand how plant traits might influence soil C storage in tropical forests.

The physical and chemical properties of organic matter inputs to forest soils are hypothesised to change during secondary forest succession, with the shift from ‘acquisitive’ to ‘conservative’ tree growth strategies, as fast‐growing, light‐demanding species are gradually replaced with slow‐growing, shade‐tolerant species (Chazdon, 2014; Whitfeld et al., 2014). Trees with acquisitive growth strategies prioritise investment in light capture and rapid growth, which generally results in low specific wood density, large specific leaf area (SLA), high foliar nutrient concentrations and lower investment in foliar defences (i.e. ‘high‐quality’ plant litter; Chazdon, 2014; Poorter et al., 2004; Wright et al., 2004). Conversely, trees with conservative growth strategies invest more resources in structure and defence, resulting in high specific wood density and tougher plant material with lower nutrient concentration (i.e. ‘low‐quality’ litter). The functional traits associated with distinct plant growth strategies also influence the decomposition of plant material (Cornwell et al., 2008), whereby litter from light‐demanding species is preferentially utilised by decomposer organisms, and therefore decays at a faster rate than litter from shade‐tolerant species (Laird‐Hopkins et al., 2017). Microbial decomposition products of ‘high‐quality’ plant litter are the main precursors of stable SOM (Cotrufo et al., 2013), and we would expect greater SOM accrual in forests with a greater proportion of light‐demanding species (typical of early succession) than in forests with comparatively more shade‐tolerant species (typical of older forests).

However, tree community composition does not always change predictably with forest age. Although succession is often conceptualised as a transition from fast‐growing species with acquisitive traits to a slow‐growing ‘climax’ community with conservative traits, successional trajectories can be influenced by former land use, soil conditions, the composition of the surrounding landscape, ongoing disturbance and seed dispersal, among other factors (Baldeck et al., 2013; Chazdon, 2014; Dent & Estrada‐Villegas, 2021; Norden et al., 2015; Turner et al., 1998). The lack of a predictable transition from light‐demanding pioneer species to shade‐tolerant late‐successional forest trees during secondary succession would be reflected in plant C inputs to the soil and could therefore explain why soil C stocks do not necessarily increase with secondary forest age. Importantly, although light‐demanding species are likely to dominate in young secondary forests, they can still play an important role throughout succession: Not only do natural disturbances create canopy gaps allowing light‐demanding species to colonise areas of old‐growth forest (Chazdon, 2014), but long‐lived pioneers can also persist in the canopy of late‐successional forests (Rüger et al., 2020). These long‐lived pioneers may be an important source of nutrient‐rich litter inputs in late‐successional secondary forests because they grow fast, but have long life spans, so they attain large stature in comparison to short‐lived pioneer species (Rüger et al., 2020). Indeed, long‐lived pioneers can dominate most successional stages and contribute substantially to above‐ground biomass through mid‐ to late secondary succession (Finegan, 1996; Gutiérrez et al., 2008; Rüger et al., 2020). The persistence of light‐demanding species during late‐successional stages could enhance SOM formation by maintaining inputs of easily decomposable plant litter, thereby increasing soil C storage.

Our study aims to improve understanding of soil C storage in secondary growth tropical forests by assessing the role of plant community composition in determining soil C accumulation. We hypothesised that tree functional composition determines soil C storage via the quality of resources available to decomposers, and that differences in tree functional composition among stands of similar age could explain the lack of a clear trajectory in soil C stocks through time. To test our hypothesis, we characterised the relationship between tree functional composition and soil C storage along a chronosequence of naturally regenerating lowland tropical forest in central Panama comprising 10 stands ranging from 40 years old to old‐growth forest (>500 years). Previous work at the site found a strong link between soil C and N stocks, but no relationship between soil C stocks and former land use or forest age (Jones et al., 2019). Thus, we investigated whether soil C stocks are instead linked to shifts in functional characteristics of the tree community. Based on preferential microbial utilisation of ‘high‐quality’ litter, we hypothesised that soil C stocks would increase with the relative proportion of light‐demanding trees in mid‐ to late‐successional tropical forest stands.

## 2 MATERIALS AND METHODS

### 2.1 Study site description

The fieldwork was carried out with the permission and support of the Smithsonian Tropical Research Institute (project #3218). The chronosequence is located in the Barro Colorado National Monument (BCNM) in Panama, Central America (9.15°, −79.85°). The climate is classified as moist tropical with a distinct dry season from January to April. Long‐term records from the weather station on Barro Colorado Island indicate a mean annual temperature of ~27°C and an average annual rainfall of ~2660 mm (Paton, 2021), of which *c*. 90% falls in the rainy season (Windsor, 1990). The chronosequence overlies a mixture of volcanic (andesite) and sedimentary (volcanic and marine) geology, supporting mainly Cambisols and Ferralsols (Baillie et al., 2007), but soil C and nutrients do not vary significantly among different soil types (Grimm et al., 2008; Jones et al., 2019; Yavitt, 2000).

**FIGURE 1:**
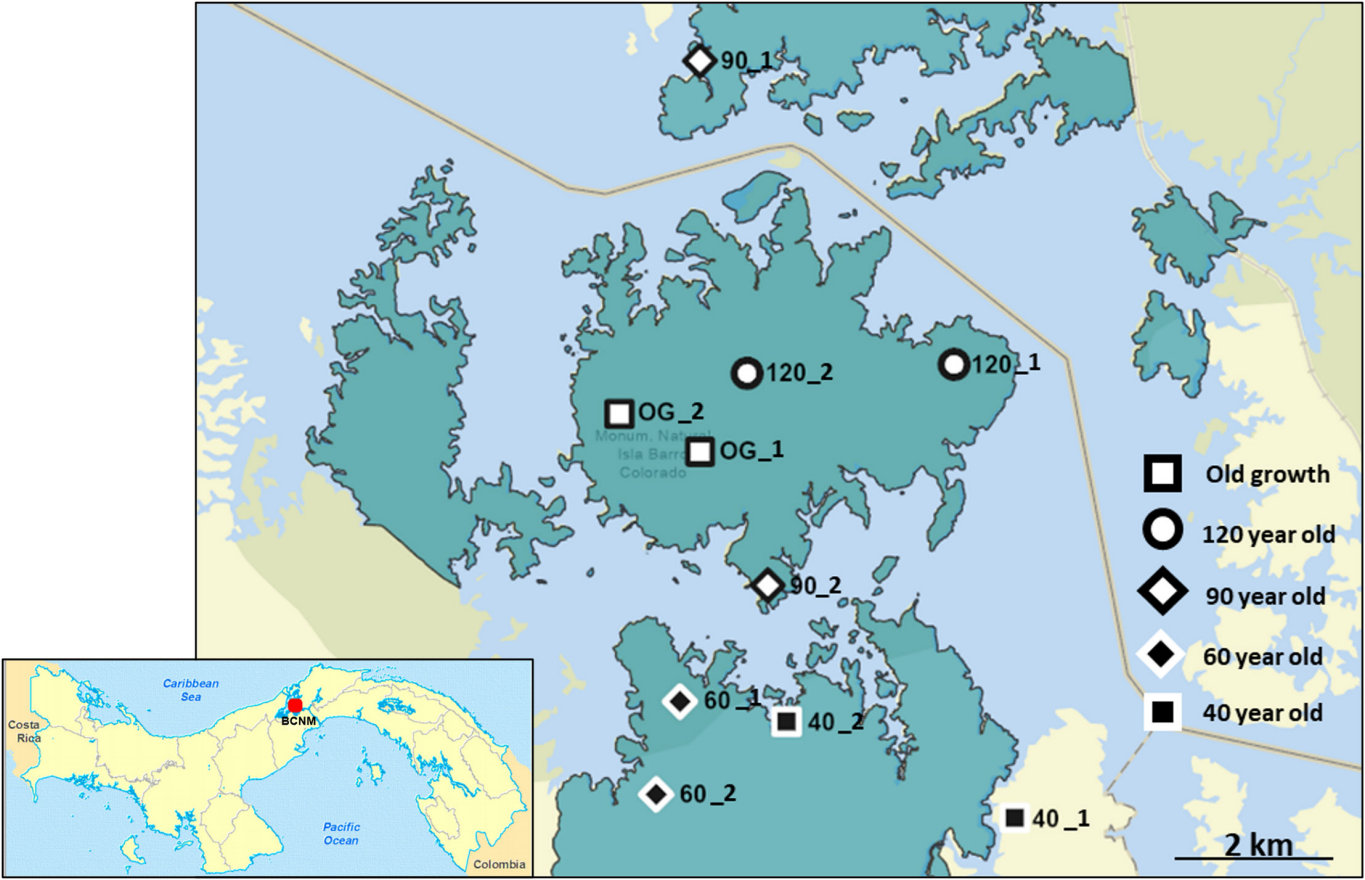
Map of the Barro Colorado Nature Monument (BCNM) in Panama, Central America, showing the approximate location of 10 stands, comprising two replicates in five age classes along a chronosequence of naturally regenerating tropical forest. Stands are labelled with the approximate stand age and the replicate number, whereby OG is old‐growth forest. Green shaded area indicates land within the BCMN (land outside BCMN shown in lighter shades), solid orange line indicates route of Panama Canal. Insert map shows location of the BCNM in Panama. Maps courtesy of STRI GIS Data Portal

The chronosequence consists of permanent plots embedded in 10 forest stands, each at least 5 ha in size (Figure 1): two stands each of four secondary forest (SF) age classes, which were *c*. 40, 60, 90 and 120 years old at the time of this study (Denslow and Guzman, 2000; Dent et al., 2013), and two old‐growth forest (OG) stands for comparison. The OG forest stands are >500 years old with no evidence of previous clearance or agriculture. The stand ages are henceforth given as: 40Y, 60Y, 90Y, 120Y and OG; the dominant tree species and a comparison of species diversity for each age category are shown in Table S1.

The chronosequence is considered representative of forest recovery following the widespread practice of swidden agriculture in the tropics, where deforestation for agriculture and subsequent farm abandonment over time has created a mosaic of OG and naturally regenerating SF (Denslow and Guzman, 2000; Dent et al., 2013). Prior to the designation of the BCNM as a nature reserve in 1977, past land use on SF stands included pasture for cattle, fruit and vegetable production and banana plantations of varying duration and intensity (Denslow and Guzman, 2000; Leigh et al., 1983). Although prior land use did not explain differences in soil C or N stocks among stands (Jones et al., 2019), historic differences in land‐use patterns among stands are acknowledged as a potential cause of differences in initial stand composition and subsequent successional processes (Denslow and Guzman, 2000; Dent et al., 2013).

### 2.2 Tree community characterisation

We used tree census data and a measure of tree community shade tolerance to characterise forest stands and assess the relationship between soil C stocks and tree functional groups during secondary succession. Stand basal area was calculated from census data collected in 2011 from two 0.16 ha transects per stand (except for one stand where only one 0.16 ha transect was censused; Dent et al., 2013). Stem diameter at breast height (DBH; 1.3 m) for each individual ≥10 cm DBH was converted to stem basal area (m^2^) and the sum of all stems in each stand was then divided by the total area to obtain a single stand‐level basal area. $\text{Stembasalarea}\left(m^{2}\right)={\left(\frac{\mathrm{DBH}\left(\mathrm{cm}\right)}{200}\right)}^{2}\times \pi$. To characterise shade tolerance of the tree communities in each stand, we used light effect categories proposed by Rüger et al. (2009), based on species‐specific growth responses to increasing light. Species were assigned to one of three growth response categories and assigned light effect values (*b*) based on sapling recruitment: ‘accelerating species’ (*b* ≥ 1), where recruitment increases with increasing light and the increase is greater at higher light levels; ‘decelerating species’ (1 > *b* ≥ 0), where recruitment increases with increasing light, but the increase is lower at higher light levels; ‘negative species’ (*b* < 0), where recruitment is higher at lower light and decreases with increasing light levels. These categories are considered comparable to the light requirements for recruitment of fast‐ and slow‐growing species (Rüger et al., 2009).

To assign each individual tree to a functional group, we used the mean light effect values of each species to classify them as ACC (accelerating), DEC (decelerating) or NEG (negative; *sensu* Rüger et al., 2009); species without light effect data were classified as ‘unknown’. These categories are henceforth referred to as tree functional groups. We then calculated an importance value (IV) for each species based on the relative frequency of individuals (Equation 1) and the relative dominance calculated from their basal area (Equation 2). Thus, the IV (Equation 3) considers species with large numbers of individuals as well as those with a large total biomass to be of equal importance for determining ecosystem processes (Lohbeck et al., 2012):
(1)

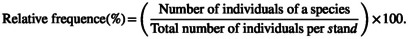



(2)

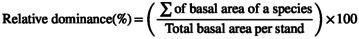

(3) $$\text{Importance value}\left(\%\right)=\frac{\text{Relative frequency}+\text{Relative dominance}}{2}$$

Finally, to provide a comparative measure of community shade tolerance across the chronosequence stands, we calculated the relative influence (RI) of the ACC, DEC and NEG tree functional groups in each stand, whereby the summed IVs for each group were expressed as a proportion of the total IV of all species per stand. Of the 276 tree species identified across the 10 stands (14,046 individual trees), light effect data were available for 200 species (12,825 individual trees). Therefore *c*. 72% of species and *c*. 91% of individuals were assigned to growth response categories (DEC = 10,965 individuals of 128 species, ACC = 1276 individuals of 61 species, NEG = 584 individuals of 11 species), which amounted to *c*. 88% of the total proportion of species expressed as RI (60% DEC, 24% ACC, 4% NEG and 12% unknown).

#### 2.2.1 Soil sampling and analysis

In each of the 10 stands, we established four (20 m × 10 m) sampling blocks, spaced at 40 m intervals along a 160‐m transect. Soil was collected at two 10‐cm depth increments from 0 to 20 cm at three points (5, 10 and 15 m) along each transect and within each sampling block using a 4.8‐cm diameter soil corer. The samples were composited by block and depth and mixed thoroughly, giving a total of 80 composite samples (10 stands × 4 blocks × 2 depth increments). The samples were stored at 4°C within 4 h of collection, sieved to <2 mm to remove debris and stones and processed for analysis within 48 h. Soil C and N concentration (mass‐based %) was measured by high‐temperature combustion gas chromatography (Vario El III C/N analyser, Elementar) using 30 mg (±1 mg) subsamples of ground, air‐dried soil. Soil C stocks (Mg ha¯^1^) were calculated for 0–10 and 10–20 cm depth increments in each stand following Equation 4:

(4) $$C_{\text{stock}}=D\times \mathrm{Bd}\times C_{\text{conc}},$$

where *D* is the depth of sample (cm), *Bd* is the mean soil bulk density (provided by Jones et al., 2019), and *C*_conc_ is the mean soil C concentration (%) from the four measured values per stand and depth increment.

### 2.3 Data analysis

Data analyses were performed using R version 3.5.2 (R Core Team, 2018) using the vegan package for multivariate analyses (Oksanen et al., 2007) and the lme4 (Bates et al., 2015) and lmerTest (Kuznetsova et al., 2017) packages for mixed effects models.

We first assessed whether our tree functional groups, assigned using the light effect value *b*, corresponded to plant functional traits associated with light‐demanding and shade‐tolerant species. We performed a principal component analysis (PCA, *rda* function in the vegan package) based on four leaf traits associated with differences in plant life‐history strategies: leaf density, leaf thickness, specific leaf area (SLA) of shade leaves and N concentration of shade leaves (Chazdon, 2014; He et al., 2019; Poorter & Bongers, 2006), which were available for 64 species at the study site (representing 23% of all species and 29% of all individuals at the study site; Wright et al., 2010; accessed from the TRY database http://www.try‐db.org). All trait values were centred and scaled before analysis and fitted to the ordination as vectors to aid interpretation (*envfit* function). We then assessed whether functional group (ACC, DEC, NEG) explained the separation of species in ordination space with a permutational multivariate analysis of variance (perMANOVA, *adonis* function; Anderson, 2017) based on Euclidean distance and 9999 permutations. We then extracted the scores for the first two principal components (PC1 and PC2) and tested their relationship with the mean *b* values of the tree species using Pearson's correlation test (*cor.test* function). Finally, we tested differences among tree functional groups for each of the four plant traits using linear models (lm function), modelling leaf N concentration, leaf thickness, leaf density or SLA as a function of tree functional group.

We assessed the relationships between soil C stocks (Mg ha¯^1^) and tree functional groups with linear mixed effects models (*lmer* function). Models were based on stand‐level mean soil C stocks calculated from the four sampling blocks, with stand identity included as a random effect. We accounted for declining soil C concentration with depth by including depth increment as a fixed effect in all models. Furthermore, given that soil C stocks were strongly related to soil N stocks (Jones et al., 2019), we included soil N stocks as a fixed covariate in all models. As the RI values of the tree functional groups within stands represent relative proportions that sum to one, we conducted a compositional analysis in which we used the additive ln‐ratio transformation for the percentage compositions, with relative influence (RI) of DEC and NEG species as numerators and (RI) ACC as the denominator in the ratios (henceforth referred to as ln(DEC/ACC) and ln(NEG/ACC)). Therefore, the fixed effects in the full model were the relative influence of tree functional groups (ln(DEC/ACC) and ln(NEG/ACC)), depth, the interaction between tree functional groups and depth and N stocks; stand was included as a random effect. We used Akaike information criterion (AIC) values to rank models and then used a likelihood ratio test to compare the top‐ranked model to the null model, which included soil depth and N stocks as fixed effects and stand as a random effect (Table S2). Hence, a significant difference of the final model to the null model indicates an influence of tree functional composition on soil C stocks over and above the established relationship with soil N (Jones et al., 2019). The significance of fixed effects was determined using Satterthwaite's approximation to estimate degrees of freedom, and the fit of the final model was checked using diagnostic plots. Results are reported as significant at *p* < 0.05; for linear mixed effects models chi‐square and *p* values are given for the comparison between the final model and the corresponding null model.

## 3 RESULTS

**FIGURE 2:**
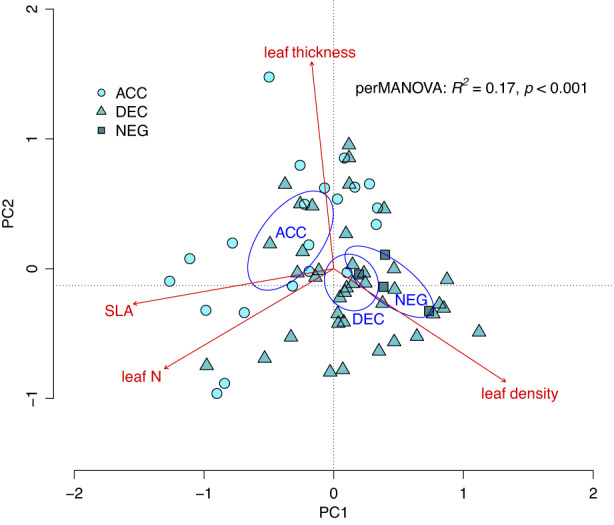
Ordination plot (principal component analysis) based on four plant functional traits, showing the functional groups for 64 tree species according to their light response effect values, where ACC = accelerating, DEC = decelerating and NEG = negative growth response to increasing light; ellipses show the separation of functional groups in ordination space based on 99% confidence limits based on standard errors; traits that explain the separation of species in ordination space are shown as vectors, where leaf N and SLA are the nitrogen concentration and specific leaf area of shade leaves, respectively.

**FIGURE 3:**
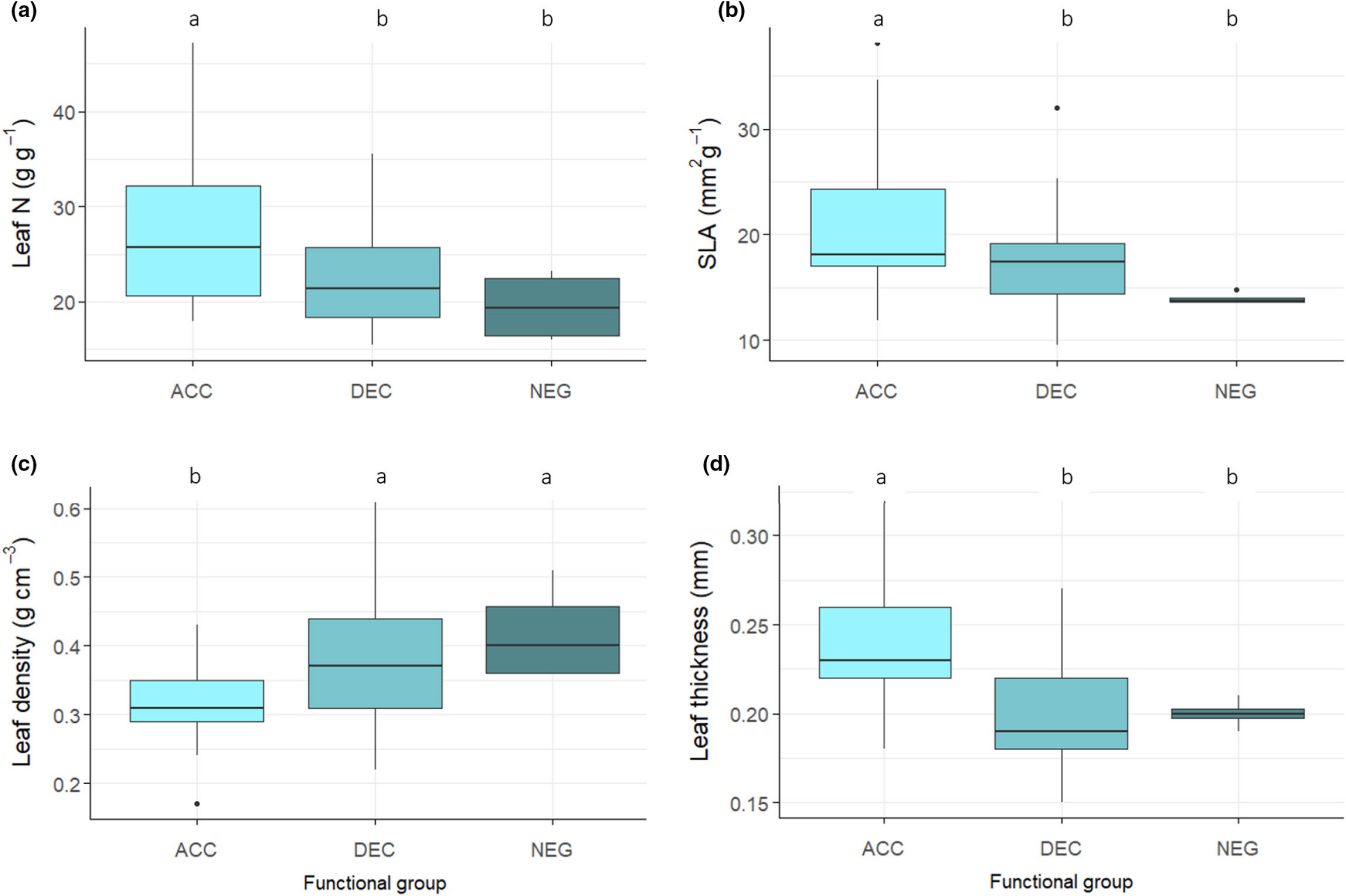
Comparison of leaf traits between accelerating growth (ACC), decelerating growth (DEC) and negative growth (NEG) tree functional groups in a lowland tropical forest in Panama, Central America, showing (a) leaf nitrogen (N) concentration of shaded leaves, (b) specific leaf area (SLA) of shaded leaves, (c) leaf density and (d) leaf thickness. Data were taken from Wright *et al*. (2010), accessed from the TRY database (http://www.try‐db.org). Boxes denote the 25th and 75th percentiles and median lines are given for ACC *n* = 21, DEC *n* = 39; NEG *n* = 4. Whiskers indicate values up to 1.5× the interquartile range and dots indicate outliers. Letters indicate significant differences between tree functional groups at *p* < 0.05.

The PCA and perMANOVA based on plant traits for the subset of 64 species demonstrated separation of species in ordination space that was related to tree functional group (*R* ^2^ = 0.17, *p* < 0.001) and the first two principal components explained 85% of the variation among species. Leaf density, leaf N and SLA were strongly correlated with PC1, which explained 50% of the variation among species, whereas leaf thickness was correlated with PC2, which explained 34% (Figure 2). The species‐specific light effect value *b* was correlated with PC1 (*r* = −0.39, *p* = 0.002; Figure S2) but not PC2, indicating that our classification based on light effect values reflects variation in three key leaf functional traits. Overall, ACC species had higher leaf N concentration (*F*_2,61_ = 5.39, *p* = 0.007), SLA (*F*_2,61_ = 5.16, *p* = 0.009) and leaf thickness (*F*_2,61_ = 5.35, *p* = 0.007), but lower leaf density (*F*_2,61_ = 9.20, *p* < 0.001) than DEC or NEG species (Figure 3).

### 3.1 Patterns in soil carbon and tree functional groups across the 10 stands

**FIGURE 4:**
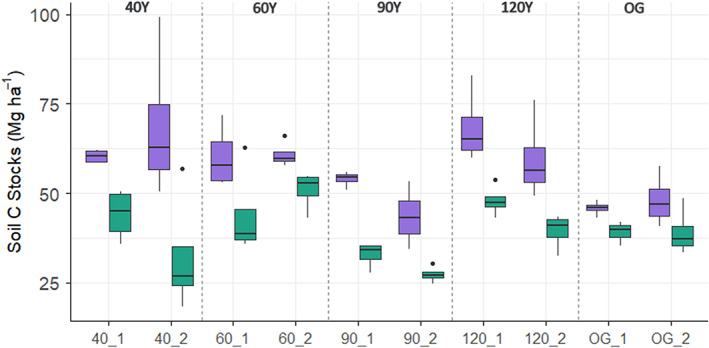
Soil carbon stocks (Mg ha^−1^) measured at two depth increments (purple = 0–10 cm, green = 10–20 cm) in each of 10 stands across an age gradient of lowland tropical forest sites in Panama, Central America, comprising 40‐, 60‐, 90‐ and 120‐year‐old stands and old‐growth (OG) forest. Stand codes on the *x*‐axis give the stand age and replicate number. Boxes denote the 25th and 75th percentiles and median lines are given for *n* = 4, whiskers indicate values up to 1.5× the interquartile range and dots indicate outliers.

**FIGURE 5:**
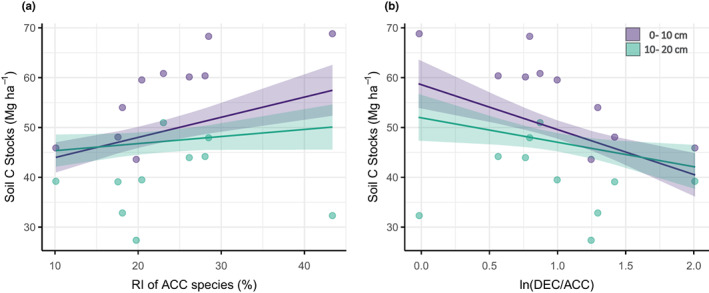
Estimated marginal mean effects (line) with 95% confidence intervals (shading) of tree functional components on soil carbon (C) stocks at two depth increments, showing (a) the relative influence (RI) values (%) of accelerating species (ACC) and (b) the ln‐ratio of the RI values for decelerating (DEC) and ACC species (ln DEC/ACC) across 10 stands of naturally regenerating lowland tropical forest in Panama Central America. In (a), a linear mixed model (LMM) was fit to allow estimates for display purposes, with the model following the same random effects structure and covariates as panel (b). The model formula and statistics for (b) are given in Table S2. Points represent stand means calculated from *n* = 4 blocks.

Across all stands, mean soil C concentration at 0–10 cm was 4.74 ± 0.15%, soil C stocks were estimated at 56.96 ± 11.92 Mg ha¯^1^ and both declined with depth, but there was no clear pattern with forest stand age (Figure 4; Table 1). Overall, the RI of DEC species tended to increase, and the RI of ACC species tended to decrease with increasing forest age up to 90 years, but the functional composition of the 120Y stands was similar to the 60Y stands and the functional composition of the OG forests was similar to the 90Y stands (Table 1). The RI of NEG species was low (<7%) in all stands (Table 1). The proportions of species not assigned to a growth response (‘unknown’ species) ranged from 4.3% to 21.6% of the total number of species per plot, but there was also no trend with forest age class (Table 1). Soil C stocks increased with the RI of ACC species (Figure 5a) and the model showed a significant negative relationship between soil C stocks and ln(DEC/ACC), but not ln(NEG/ACC) (*p* = 0.0014; Figure 5b; Figure S1). The relationship between soil C and tree functional composition was only apparent at 0–10 cm depth (ln(DEC/ACC) × depth interaction *p* = 0.014; Figure 5). Hence, the best model explaining the change in soil C stocks across stands included the ln‐ratios for the tree functional groups, sampling depth and soil N stocks (*χ* ^*2*^ = 15.26, *p* = 0.004; Table S2; Figure 5b).

**TABLE 1:** Soil properties and tree functional groups for 10 stands along a chronosequence of naturally regenerating tropical forest in Panama, Central America, comprising 40‐, 60‐, 90‐ and 120‐year‐old stands and old‐growth (OG) forest, showing soil carbon (C) and nitrogen (N) concentrations (%) and stocks (Mg ha^−1^), soil C:N ratio at two depths (0–10 and 10–20; means given for *n* = 4 per stand) and the relative influence (RI) of accelerating growth species (ACC), decelerating growth species (DEC), negative growth species (NEG), the ratio of DEC:ACC (DEC:ACC) species and the RI of species with unknown growth response (unknown). Stand codes give the stand age in years and replicate number

Stand code (age_rep)		40Y_1	40Y_2	60Y_1	60Y_2	90Y_1	90Y_2	120Y_1	120Y_2	OG_1	OG_2
**Soil C %**	0–10 cm	4.56	5.22	4.81	5.80	4.51	3.76	4.83	5.60	3.99	4.37
	10–20 cm	2.50	2.18	3.05	3.23	2.12	1.81	2.91	2.84	2.69	2.79
**Soil C (Mg ha** ^**−1**^ **)**	0–10 cm	60.37	68.82	60.14	60.84	54.00	43.60	68.29	59.55	45.89	48.0
	10–20 cm	44.18	32.31	43.95	50.96	32.84	27.36	47.94	39.49	39.20	39.10
**Soil N %**	0–10 cm	0.38	0.42	0.44	0.49	0.39	0.26	0.42	0.44	0.40	0.38
	10–20 cm	0.19	0.12	0.24	0.23	0.14	0.06	0.24	0.19	0.25	0.22
**Soil N (Mg ha** ^**−1**^ **)**	0–10 cm	4.97	5.54	5.44	5.09	4.67	3.04	5.91	4.68	4.55	4.21
	10–20 cm	3.27	1.82	3.46	3.59	2.09	0.95	4.00	2.61	3.62	3.08
**RI tree functional groups (%)**	ACC	28.1	43.4	26.2	23.0	18.1	19.8	28.5	20.4	10.1	17.6
	DEC	49.3	42.7	56.2	55.1	66.1	68.8	63.1	55.4	75.2	72.6
	NEG	1.03	0.2	6.9	1.8	1.5	7.2	1.7	6.1	5.0	4.3
	DEC:ACC	1.8	1.0	2.2	2.4	3.7	3.5	2.2	2.7	7.5	4.1
	‘Unknown’	21.6	13.7	10.8	20.1	14.3	4.3	6.7	18.1	9.7	5.5

## 4 DISCUSSION

Our study demonstrates that tree functional composition plays a key role in the storage of soil C during secondary tropical forest succession, and thus provides a compelling explanation for the lack of a clear trajectory of increasing soil C stocks over time. Along our chronosequence, surface soil C stocks were strongly related to the relative influence of ACC trees. As the separation of tree functional groups in ordination space was explained by leaf traits associated with light‐ versus shade‐tolerance as well as decomposition rates, we propose that the relationship between soil C stocks and the relative influence of ACC species can be attributed to the quality of resources available to decomposer organisms. Thus, our findings conform to recent theoretical frameworks and empirical studies demonstrating preferential stabilisation of compounds from N‐rich plant material in the soil during microbial decomposition, resulting in greater SOM accrual (Castellano et al., 2015; Cotrufo et al., 2013; Haddix et al., 2016).

### 4.1 Tree functional composition as a driver of soil C storage

Tree functional composition might influence soil C accumulation during secondary succession because soil C storage is determined by the balance between inputs and losses of organic C during decomposition (Laganière et al., 2010; Post & Kwon, 2000), and microbial decomposition of plant litter is largely determined by plant traits (Bakker et al., 2011; Meier & Bowman, 2008). We hypothesised that differences in tree functional composition among stands would influence soil C storage via plant traits representing resource quality for microbial decomposers. The tree functional groups used in this study correspond largely to previous definitions of light‐demanding or pioneer species (ACC) and shade‐tolerant or climax species (DEC; Dent et al., 2013). In our study, ACC species were associated with higher SLA and leaf N concentration, whereas DEC and NEG species were associated with lower leaf N and higher leaf density (Figures 2 and 3), which is consistent with the fast–slow continuum at the leaf level (Reich, 2014; Rüger et al., 2018). Thus, although the ACC, DEC and NEG classifications relate primarily to tree demography (Rüger et al., 2009), they also act as a proxy for acquisitive versus conservative life‐history strategies. Light‐demanding or ACC species are associated with ‘competitor’ traits (Grime, 1974), which contribute to rapid growth rates and photosynthetic capacity. By contrast, the leaf traits of DEC and NEG species indicate a more conservative resource‐use strategy (Figures 2 and 3).

The leaf traits separating ACC and DEC and NEG along the first axis in ordination space in our study (SLA, leaf N concentration and leaf density) are often strongly related to decomposition rates (Bakker et al., 2011) because they represent high‐ versus low‐quality resources to microbial decomposers (Cornwell et al., 2008; Santiago, 2007). The significant relationship between the first ordination axis and the light effect value *b* thus suggests that trait differences among our broad functional groups are likely to influence C turnover and soil C storage. Light‐demanding species typical of early successional stages can supply large amounts of nutrient‐rich litter to the soil which are rapidly decomposed (Bakker et al., 2011; Coley, 1983; Szefer et al., 2017). Conversely, shade‐tolerant DEC or NEG species produce longer lived, but low‐quality litter (Grime et al., 1996), which reduces decomposition rates (Kutsch et al., 2009). These differences in plant litter decomposition drive soil organic matter (SOM) formation, because the products of successive microbial processes during decomposition increase C stability in SOM through aggregation and chemical bonding in the soil matrix (Castellano et al., 2015; Cotrufo et al., 2013; Cotrufo et al., 2015, 2019; Faucon et al., 2017; Hatton et al., 2015; Lavallee et al., 2020; Schmidt et al., 2011). The availability of N plays a key role in the formation and of SOM because the initial C:N ratio of plant litter is higher than the C:N ratio of the microbial biomass and thus microbial activity is constrained by N availability (e.g. Billings & Ballantyne, 2013; Hessen et al., 2004). Consequently, rapid turnover of labile plant substrates that are rich in N can increase SOM stabilisation and storage in soils that are not yet C saturated (Castellano et al., 2015; Faucon et al., 2017). We demonstrate that soil C stocks were highest in stands with a greater relative influence of light‐demanding ACC tree species (Figure 5), of which many have ‘high‐quality’ litter (Bakker et al., 2011; Figure 3), representing a substantial source of labile C compounds and nutrients for soil microbes. This major role of leaf litter decomposition in tropical forest soil carbon storage (Sayer et al., 2021) would also explain why we only observed a significant relationship between soil C stocks and tree functional composition at the soil surface (0–10 cm; Figure 5a). Thus, our results suggest that high‐quality plant inputs may play a key role in soil C accumulation during secondary succession in these forests.

### 4.2 Successional trajectories of tree community functional groups during forest recovery

In theory, high abundance of fast‐growing ACC species in the early stages of succession would contribute to rapid accumulation of soil C, which attenuates as they are eventually replaced by slow‐growing DEC and NEG species. At our study sites, soil C stocks increased between 40‐ and 60‐year‐old stands (Table 1), which likely reflects the high abundance of ACC species during early to mid‐succession, whereas the continued influence of ACC species during later succession can be attributed to the persistence of long‐lived pioneers, which form a significant proportion of the canopy in secondary tropical forests (Rüger et al., 2020). For example, *Gustavia superba* (Kunth) O.Berg, a long‐lived pioneer species (Rüger et al., 2020) made up >50% of individuals in the canopy of one of the 120Y stands (120_1; Dent et al., 2013), which also had the second‐highest C stocks measured in this study (Table 1; Table S1).

Differences in tree functional composition could explain why several meta‐analyses and syntheses have reported either a weak relationship, no significant change or contrasting results for changes in soil C as a function of forest age (Li et al., 2012; Marín‐Spiotta & Sharma, 2013; Martin et al., 2013; Powers & Marín‐Spiotta, 2017; Yang et al., 2011). It is conceivable that the lack of consistency among studies can be attributed to differences in the dominant tree functional group (and associated organic matter quality), which is not necessarily predictable with forest age. Despite expectations that light‐demanding species are replaced by shade‐tolerant trees during forest regrowth, the successional trajectory of tree functional composition can be influenced by a multitude of factors, including soil physicochemical properties and former land use (Chazdon, 2014). For example, Dent et al. (2013) report that the high proportion of shade‐tolerant species they observed in one of the youngest stands (40_1) may in part be due to a shorter farming history and therefore lower soil disturbance than stands farmed for longer periods. In addition, disturbances such as storms can create gaps and promote recruitment of light‐demanding tree species in late‐successional stages (Chazdon, 2014) and as such, the functional characteristics of dominant tree species may differ markedly among secondary forest stands of the same age (Boukili & Chazdon, 2017; Norden et al., 2015). Thus, successional trajectories and disturbances that create conditions favourable for the persistence of pioneer species could have profound effects on soil C accumulation and storage in tropical forests. Characterising tree communities in secondary tropical forests by functional groups rather than successional age formed a key component of our study because it considers both the quality of organic inputs from different tree functional types and the pathways of C stabilisation in soils. Considering such plant–soil interactions revealed clear relationships between tree community composition and soil C stocks during secondary succession. We believe that similar characterisation of tree community composition could elucidate patterns of soil C accrual during secondary succession in many other tropical forests.

It is important to note that previous work along the same chronosequence of stands demonstrated increasing community‐level shade tolerance with stand age (Dent et al., 2013). However, the study by Dent et al. (2013) was based on data collected in 1994, when the youngest stands were still at an early successional stage, with high abundances of ACC species. Here, we assessed mid‐ to late‐successional stages and used a different measure of dominance, based on species importance values, to calculate the relative influence (RI) of each functional group. We expected that the use of RI values in our study was more likely to reveal relationships between tree communities and soil C stocks as they assume that many small individuals are as important for determining ecosystem processes as a small number of large trees (Lohbeck et al., 2012). Given the clear relationship between soil C stocks at 0–10 cm depth and the RI values for ACC species (Figure 5a), our approach suggests that the influence of tree functional groups on soil processes does not necessarily follow widely observed patterns in tree species abundance or basal area during secondary forest succession. For example, despite the decline in abundance of light‐demanding pioneer species with successional age (Dent et al., 2013), the high relative influence of ACC species in our study (Table 1) suggests that long‐lived pioneer species in the later stages of succession may still make a substantial contribution to soil C storage. Therefore, the influence of tree functional groups on soil processes may not be as readily apparent when species abundance data are used.

There is potential for our approach to be extended by expanding the functional classification of trees using additional traits that are relevant to SOM formation and stabilisation. The light effect value we used here is based on a single‐trait axis relevant to demographic processes (the ‘growth‐survival’ trade‐off; Rüger et al., 2009), which we used as a proxy for acquisitive versus resource‐conservative life‐history strategies. By focusing on tree species' life‐history trade‐offs that influence soil microbial processes, we may be able to identify similar trait axes that more accurately represent the influence of plants on processes such as SOM formation and soil C storage. Given the paucity of trait data for tropical trees, we were only able to consider relevant leaf traits for a subset of our species, but inclusion of root traits and turnover rates would likely provide additional explanatory power, and perhaps elucidate patterns of soil C storage at greater soil depths. Thus, our study suggests that targeted trait collection could advance our understanding of how functional diversity influences ecosystem carbon storage in tropical forests.

### 4.3 Indirect influence of former land use on tree functional composition and soil C storage

Differences in tree functional composition might also explain the influence of former land use on soil C accumulation during succession, and why soil C stocks do not increase consistently with stand age (Martin et al., 2013; Orihuela‐Belmonte et al., 2013). Former land use not only affects soil C stocks through the extent of C loss from initial soil disturbance (Don et al., 2011; Guo and Gifford, 2002), but also influences the recruitment and growth of tree species in the early stages of succession. The intensity and duration of previous soil disturbance can affect both the viability of the soil seed bank (e.g. due to soil compaction) and the germination success of particular species (e.g. the requirement of exposed soil for some small‐seeded pioneers, Chazdon, 2003; Guariguata & Ostertag, 2001). Thus, if former land use influences species recruitment during the early stages of secondary succession, differences in tree community structure would mediate the relationship between prior land use and soil C stocks, explaining why it is observed in some studies but not others. Our findings also have implications for forest restoration and management decisions. Light‐demanding tree species are commonly included in forest restoration strategies as they grow rapidly creating an environment that allows slow‐growing, shade‐tolerant species to establish (Mayer et al., 2020). This initial colonisation by fast‐growing, light‐demanding tree species should also result in rapid soil C accumulation in the early stages of forest restoration, when site conditions are favourable (Powers & Marín‐Spiotta, 2017). Conversely, commercial plantations of tropical hardwoods, which are often late‐successional species, could decelerate accumulation of soil C stocks. Our results suggest that soil C storage could be enhanced by management or restoration practices that ensure the persistence of long‐lived pioneer species, for example by informed choice of ‘nurse trees’. Hence, understanding the influence of light‐demanding tree species on soil C storage not only can help us improve predictions of C sequestration in naturally regenerating secondary forests but might also have wider relevance for forest restoration.

## 5 CONCLUSIONS

We demonstrate that tree functional groups explain differences in soil C stocks during secondary tropical forest succession, which likely explains the lack of a clear trajectory of increasing soil C with successional age. We propose that the broad distinctions between light‐demanding, accelerating growth (ACC) species and shade‐tolerant, decelerating growth (DEC) species and their respective relative influence on soil C storage provides the basis for a useful, quantitative method to investigate how disturbance and successional dynamics in tree communities influence ecosystem scale C dynamics in recovering secondary tropical forests, whereby decomposition of N‐rich plant material results in higher C stocks in the surface soil of forests with a greater relative influence of light‐demanding (ACC) species.

Our study demonstrates a link between above‐ground plant community and below‐ground characteristics, showing that species composition in natural successional trajectories and planting choices in active restoration can influence both above‐ and below‐ground carbon accumulation. Given the global importance of secondary tropical forests for C sequestration and climate change mitigation, existing long‐term monitoring data and global plant trait databases could be used to investigate whether soil C stocks are related to plant traits and tree functional groups across other forest sites world‐wide.

## AUTHOR CONTRIBUTIONS

Emma Sayer, Lindsay F. Banin, Daisy H. Dent, Ute Skiba and Abby Wallwork conceived the ideas and designed methodology; Abby Wallwork collected the data; Abby Wallwork, Emma Sayer and Lindsay F. Banin analysed the data; Abby Wallwork and Emma Sayer led the writing of the manuscript. All authors contributed critically to the drafts and gave final approval for publication.

## CONFLICT OF INTEREST

Emma Sayer is a Senior Editor of *Functional Ecology* but took no part in the peer review and decision‐making processes for this article. There are no conflicts of interest.

## Supporting information


Figure S1
Figure S2
Table S1
Table S2

## Data Availability

Data for this study are available on figshare (Wallwork et al., 2022) with the identifier https://doi.org/10.6084/m9.figshare.21369822.
