# Supplementary material for: Soil carbon storage is related to tree functional composition in naturally regenerating tropical forests
Source: Funct Ecol. 2022 Nov 10;36(12):3175–87. doi: 10.1111/1365-2435.14221 (PMC10099939; doi:10.1111/1365-2435.14221)
Supplement: Supplementary file 1 — Figure S1 Figure S2 Table S1 Table S2 [file FEC-36-3175-s001.docx]

Soil carbon storage is related to tree functional composition in naturally regenerating tropical forests

ABBY WALLWORK ^1,2^, LINDSAY F. BANIN ^2^, DAISY H. DENT ^3,4^, UTE SKIBA ^2,^ EMMA SAYER ^1,4^

*^1^Lancaster Environment Centre, Lancaster University, UK, ^2^UK Centre for Ecology & Hydrology, Penicuik, UK, ^3^University of Stirling, Stirling, UK, ^4^Smithsonian Tropical Research Institute, Balboa, Ancón, Panama, Rep. of Panama.*

## Supplementary Tables and Figures

**Table S1:** Stand characteristics for 10 forest stands (two stands in each of five age classes; 40, 60, 90, 120-year-old secondary forest and old-growth (OG) forest) in Panama, Central America. Stand code refers to stand age in years and replicate number. Subscript numbers indicate the data source, where 1 = Denslow and Guzman (2000) and 2 = Dent, DeWalt and Denslow (2013).

| Stand code | Previous land use_1_ | Species diversity  (Fisher’s α)_2_ | Five most abundant (adult) tree species_2_ | Proportional abundance_2_ |
| --- | --- | --- | --- | --- |
| **40Y_1** | Pasture/swidden | 33.8 | *Alseis blackiana*  *Dialium guianense*  *Heisteria concinna*  *Gustavia superba*  *Malouetia guatemalensis* | 0.096  0.067  0.063  0.054  0.038 |
| **40Y_2** | Pasture/swidden | 11.4 | *Inga sapindoides*  *Apeiba tibourbou*  *Annona spraguei*  *Spondias radlkoferi*  *Gustavia superba* | 0.223  0.138  0.106  0.069  0.053 |
| **60Y_1** | Plantation | 28.6 | *Spondias radlkoferi*  *Cordia alliodora*  *Gustavia superba*  *Protium panamense*  *Oenocarpus mapora* | 0.075  0.053  0.053  0.053  0.049 |
| **60Y_2** | Pasture/swidden | 18.7 | *Inga mucuna*  *Lonchocarpus heptaphyllus*  *Miconia argentea*  *Annona spraguei*  *Inga thibaudiana* | 0.117  0.093  0.083  0.078  0.068 |
| **90Y_1** | Pasture/swidden/agriculture | 16.3 | *Protium panamense*  *Astrocaryum standleyanum*  *Gustavia superba*  *Trophis racemosa*  *Alseis blackiana* | 0.304  0.134  0.069  0.041  0.037 |
| **90Y_2** | Swidden | 25.2 | *Trichilia tuberculata*  *Apeiba membranacea*  *Protium panamense*  *Alseis blackiana*  *Tetragastris panamensis* | 0.104  0.063  0.063  0.059  0.050 |
| **120Y_1** | Pasture | 14.9 | *Gustavia superba*  *Oenocarpus mapora*  *Astrocaryum standleyanum*  *Luehea seemannii*  *Protium panamense* | 0.531  0.068  0.052  0.042  0.026 |
| **120Y_2** | Pasture | 24.8 | *Oenocarpus mapora*  *Trichilia tuberculata*  *Hirtella triandra*  *Gustavia superba*  *Alseis blackiana* | 0.130  0.102  0.065  0.046  0.037 |
| **OGY_1** | Old growth | 27.2 | *Oenocarpus mapora*  *Trichilia tuberculata*  *Protium panamense*  *Tetragastris panamensis*  *Alseis blackiana* | 0.221  0.060  0.047  0.043  0.034 |
| **OGY_2** | Old growth | 22.5 | *Oenocarpus mapora*  *Tetragastris panamensis*  *Alseis blackiana*  *Garcinia intermedia*  *Trichilia tuberculata* | 0.190  0.136  0.095  0.058  0.058 |

**Table S2:** Comparison of linear mixed effects models testing the influence of tree functional groups and soil depth on soil carbon (C) stocks (Mg ha^-1^), with soil nitrogen (N) stocks as a covariate. Statistics are given for linear mixed effects models (*lmer* function) constructed using the lme4 package (Bates *et al.*, 2015) in R version 3.5.2 (R Core Team, 2018); the degrees of freedom (df) and Akaike Information Criterion (AIC) are given for each model, and the model fit statistics (*χ*^2^ and p-value) from likelihood ratio tests of the comparison to the null model are shown; the best-fit model for each response variable is shown in bold type.

| Model | Response variable | Fixed effects | Random effects | df | AIC | *χ*2 | p-value |
| --- | --- | --- | --- | --- | --- | --- | --- |
| 1a | Soil C stocks (Mg^-1^) | log(RI NEG/RI ACC)* depth + log(RI DEC/RI ACC)* depth + N | Stand | 9 | 100.28 | 16.29 | 0.006 |
| **1b** | **Soil C stocks (Mg^-1^)** | log(RI NEG/RI ACC)+ depth + log(RI DEC/RI ACC)* depth + N | **Stand** | **8** | **99.31** | **15.26** | **0.004** |
| **1c** | Soil C stocks (Mg^-1^) | log(RI NEG/RI ACC)* depth + log(RI DEC/RI ACC)+ depth + N | Stand | 8 | 99.35 | 15.22 | 0.004 |
| 1d | Soil C stocks (Mg^-1^) | log(RI NEG/RI ACC)+ depth + log(RI DEC/RI ACC)+ depth + N | Stand | 7 | 104.04 | 8.53 | 0.036 |
| 1Null | Soil C stocks (Mg-1) | 1 * depth + N | Stand | 4 | 106.57 |  |  |


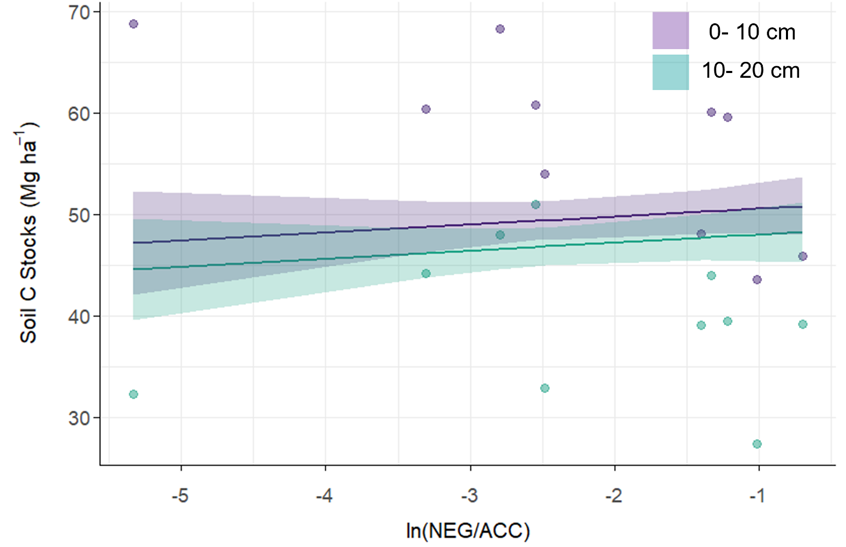


**Figure S1.** Estimated marginal mean effects (line) with 95% confidence intervals (shading) of tree functional components on soil carbon (C) stocks at two depth increments, showing the ln-ratio of the RI values for negative (NEG) and ACC species (ln NEG/ACC) across 10 stands of naturally regenerating lowland tropical forest in Panama Central America. Points represent stand means calculated from *n* = 4 blocks.


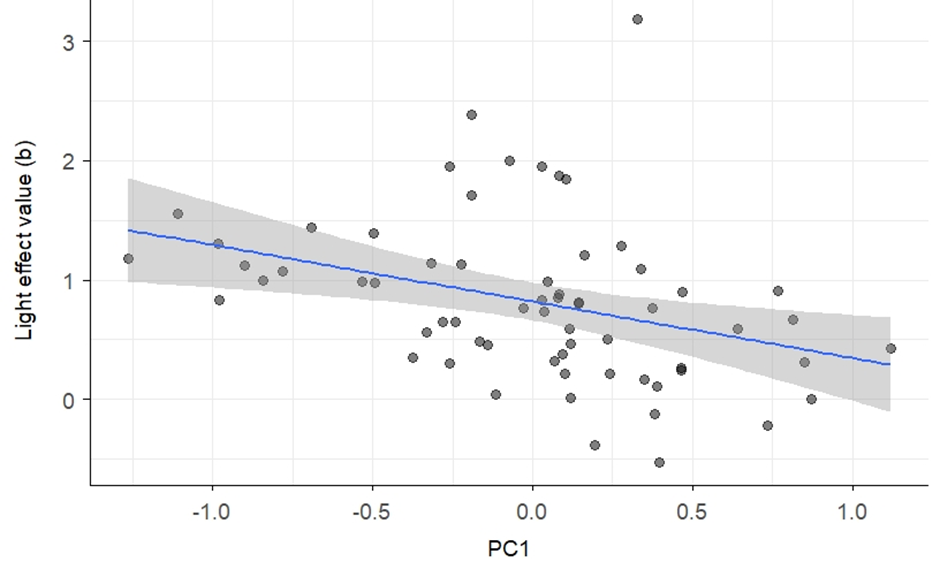


**Figure S2.** Correlation between the light effect value (b) and the scores for the first principal component (PC1) (generated from principal component analyses; PCA) based on four leaf traits associated with differences in plant life history strategies: leaf density, leaf thickness, specific leaf area (SLA) of shade leaves, and N content of shade leaves for 64 tree species present across 10 stands of naturally regenerating lowland tropical forest in Panama Central America. Of the 64 species, 21 = accelerating growth species (ACC), 39 = decelerating growth species (DEC), and 4 = negative growth species (NEG). Shaded area denotes 95% confidence intervals derived from Pearson’s correlation test.

## Supplementary references

Bates, D. *et al.* (2015) ‘Fitting linear mixed-effects models using lme4’, *Journal of Statistical Software*, 67(1). Available at: https://doi.org/10.18637/jss.v067.i01.

Denslow and Guzman (2000) ‘Variation in stand structure, light and seedling abundance across a tropical moist forest chronosequence, Panama’, *Journal of Vegetation Science*, 11(2), pp. 201–212. Available at: https://doi.org/10.2307/3236800.

Dent, D.H., DeWalt, S.J. and Denslow, J.S. (2013) ‘Secondary forests of central Panama increase in similarity to old-growth forest over time in shade tolerance but not species composition’, *Journal of Vegetation Science*, 24(3), pp. 530–542. Available at: https://doi.org/10.1111/j.1654-1103.2012.01482.x.

R Core Team (2018) ‘R: A language and environment for statistical computing’. Vienna, Austria: R Foundation for Statistical Computing. Available at: http:///www.R-project.org/.
